# The ubiquitin–proteasome system in regulation of the skeletal muscle homeostasis and atrophy: from basic science to disorders

**DOI:** 10.1186/s12576-020-00768-9

**Published:** 2020-09-16

**Authors:** Yasuo Kitajima, Kiyoshi Yoshioka, Naoki Suzuki

**Affiliations:** 1 Department of Muscle Development and Regeneration, Institute of Molecular Embryology and Genetics, Kumamoto University, 2-2-1 Honjo, Kumamoto, 860-0811 Japan; 2 Institute for Research On Productive Aging (IRPA), #201 Kobe hybrid business center, Minami-cho 6-7-6, Minatojima, Kobe 650-0047 Japan; 3 Department of Neurology, Tohoku University School of Medicine, 1-1 Seiryo-machi, Aoba-ku, Sendai 980-8574 Japan; 4 Department of Neurology, Shodo-Kai Southern Tohoku General Hospital, 1-2-5, Satonomori, Iwanuma, Miyagi 989-2483 Japan

**Keywords:** Ubiquitin proteasome system, Muscle homeostasis, Muscle stem cell, Myopathy, Muscular dystrophy, Cachexia, Amyotrophic lateral sclerosis

## Abstract

Skeletal muscle is one of the most abundant and highly plastic tissues. The ubiquitin–proteasome system (UPS) is recognised as a major intracellular protein degradation system, and its function is important for muscle homeostasis and health. Although UPS plays an essential role in protein degradation during muscle atrophy, leading to the loss of muscle mass and strength, its deficit negatively impacts muscle homeostasis and leads to the occurrence of several pathological phenotypes. A growing number of studies have linked UPS impairment not only to matured muscle fibre degeneration and weakness, but also to muscle stem cells and deficiency in regeneration. Emerging evidence suggests possible links between abnormal UPS regulation and several types of muscle diseases. Therefore, understanding of the role of UPS in skeletal muscle may provide novel therapeutic insights to counteract muscle wasting, and various muscle diseases. In this review, we focussed on the role of proteasomes in skeletal muscle and its regeneration, including a brief explanation of the structure of proteasomes. In addition, we summarised the recent findings on several diseases and elaborated on how the UPS is related to their pathological states.

## Introduction

The skeletal muscle mass accounts for approximately 40% of the total human body weight, making it the largest tissue mass present in the body [1]. Maintaining muscle homeostasis is essential for preserving the body’s integrity and activities of daily living, and thus, muscle loss or impairment is associated with several diseases, which ultimately leads to a poor quality of life. Skeletal muscle is highly plastic tissue, and its mass can change dynamically. Muscle atrophy is caused by an imbalance in proteostasis; during muscle atrophy, protein degradation overwhelms protein synthesis, leading to loss of muscle mass and muscle weakness. Paradoxically, in general, proteolysis is critical for preventing cellular dysfunction and the progression of diseases, causing complexity of proteolysis in skeletal muscle.

Perhaps the ubiquitin–proteasome system (UPS) is the most well-known cellular proteolytic system, which is responsible for degrading majority of the misfolded or defective cellular proteins [2]. Most proteins undergo degradation by being the target of the 26S proteasome through covalent attachment of a multi-ubiquitin chain. The ubiquitination of proteins involves the action of the E1 ubiquitin-activating enzyme, E2 ubiquitin-conjugating enzymes, and E3 ubiquitin–protein ligases. These tagged proteins are then recognised by the 26S proteasome, consisting of a central barrel-shaped 20S core associated with two 19S regulatory subunits [3, 4]. The latter subunits recognise and bind to the ubiquitinated proteins and initiate the adenosine triphosphate (ATP)-dependent degradation process within the catalytic core [3]. Using such a mechanism above, the UPS performs substrate-specific proteolysis.

Although there remains a lot to understand how the UPS recognises misfolded or defective proteins, disruption of the UPS is associated with pathological states, highlighting the importance of this system in cellular and whole-body homeostasis. Accordingly, several reports have demonstrated a relationship between proteasome system and lifespan. Proteasome activity has been reported to decrease with age in the brain [5], liver [6, 7], heart [8], and skeletal muscles [9], causing age-associated deteriorations. On the other hand, the genetical activation of the proteasome in yeast and Caenorhabditis elegans show protective effect against cellular aging and prolonged lifespan [10, 11].

It has also been shown that the overexpression of proteasome subunits in yeast and Caenorhabditis elegans results in an increase in the proteasome activity and leads to a prolonged lifespan [10, 11]. Conversely, transgenic mice constitutively expressing the β5t subunit of the proteasome showed reduced proteasome activity and a shorter lifespan [12]. Moreover, the loss of function of the Rpn11 subunit of the proteasome results in decreased proteasome activity, accumulation of ubiquitinated proteins, and a shortened lifespan [13]. These previous studies suggest that continuous clearance of misfolded proteins mediated by the UPS is necessary for healthy aging. We recently reported that skeletal muscle-specific reduction in proteasome activity is associated with a shortened lifespan in mouse models [14, 15]. Thus, the UPS in skeletal muscle, which comprises the largest mass in the body, may have a strong impact on aging, longevity, and whole-body homeostasis.

Proteolysis in skeletal muscle has dual nature: proteolytic pathways play a substantial physiological role in muscle atrophy, while their inhibition also promotes muscle dysfunction and weakness [16]. Indeed, contrary to the protective role of the UPS in maintaining cellular functions, many studies also have implicated that the UPS promotes muscle wasting, myofibre degeneration, and muscle weakness. Skeletal muscles function as a storage place for amino acids, and it might be one reason for the disuse atrophy. These contradictions result in variable effects of inhibitions of UPS in atrophies. Therefore, better understanding of the pathogenic role of this proteolytic system in skeletal muscle may provide novel therapeutic insights to counteract the UPS-associated diseases.

In this manuscript, we reviewed published important articles about UPS in the sequence of molecules, myotubes, skeletal muscles, until muscle atrophy in the patients. We focussed on the role of the UPS in skeletal muscle, by outlining the results of our recent studies on proteasomes in skeletal muscle and muscle stem cells. We further summarised recent findings on several diseases, and elaborated on how the UPS is related to their pathological states.

## Structure of the 26S proteasome

**Fig. 1:**
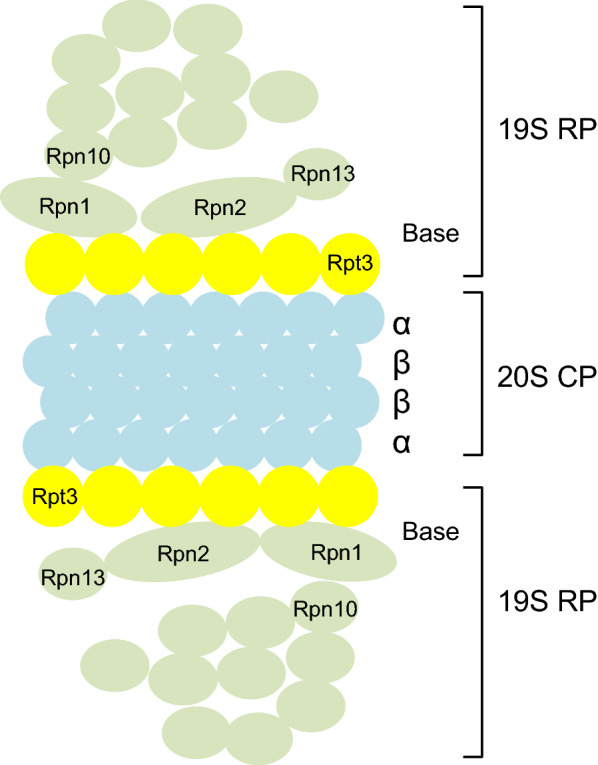
The 26S proteasome is composed of one proteolytically active cylinder-shaped particle (the 20S proteasome) and ATPase-containing complexes (the 19S cap complexes). The 19S cap complex unfolds ubiquitin-conjugated proteins to allow their entry into the 20S cylindrical particle. The 19S regulatory particles on the ends of the 20S proteasome are composed of at least 18 subunits. The base contains the two largest subunits of the proteasome, Rpn1 and Rpn2, the ubiquitin receptor Rpn10 and Rpn13, and the six ATPases, Rpt1-Rpt6. 19S RP, 19S regulatory particle. 20S CP, 20S core particle

Proteolysis by the UPS is mainly performed through a series of complex structures. The rapid degradation of ubiquitinated proteins by the 26S proteasome involves multiple enzymatic and non-enzymatic steps. The 26S proteasome is a multi-catalytic protease localised both in the nucleus and cytoplasm. As shown in Fig. 1, it is composed of one proteolytically active cylinder-shaped particle (the 20S proteasome), and two ATPase-containing complexes (known as the 19S cap complexes) [3, 4, 17]. The 19S cap complexes unfold the ubiquitin-conjugated proteins, allowing them to enter into the 20S core particle. The 20S core is composed of inner α-rings and outer β-rings, each of them has seven structurally similar subunits, respectively; α1–7 and β1–7 [18]. In particular, β1, β2, and β5 subunits display caspase-like, trypsin-like, and chymotrypsin-like proteasome activity, respectively [19, 20]. Also, these β subunits contain immunoproteasomes, in which β1, β2, β5 subunits are replaced with β1i (LMP2), β2i (MECL1), β5i (LMP7) subunits, respectively. Recently, an association between the immunoproteasome and disease has been reported and is described in Sect. 4.2.1. The 19S regulatory particles are composed of at least 18 subunits [3, 4]. Two components form the 19S proteasome, the lid and the base. The lid, which is responsible for the recognition of the polyubiquitin signal [21, 22], is composed of nine subunits, Rpn3, Rpn5-9, Rpn11-12, and Rpn15. The base is composed of the two largest subunits of the proteasome, Rpn1 (PSMD2) and Rpn2 (PSMD1), the ubiquitin receptor Rpn13, Rpn10 (PSMD4), and six ATPases, Rpt1–Rpt6 (PSMC2, PSMC1, PSMC4, PSMC6, PSMC3, PSMC5, respectively). These subunits form a large family of proteins with a highly conserved ATPase domain [23]. Rpt3, also known as PSMC4, is an essential subunit of the 26S proteasome [23] and is required for the degradation of most proteasomal substrates. The ubiquitin receptor Rpn10 attaches to Rpn1, although this association is stabilised by presence of Rpn2 [24]. Rpn2 acts as a receptor of the ubiquitin receptor Rpn13 [25–27].

## Protein degradation in the skeletal muscle

### E3 ubiquitin ligases and UPS in skeletal muscle

Muscle atrophy is a serious problem that limits the daily activities and reduces the quality of life. Regulation of the skeletal muscle mass is highly dependent on both the protein synthesis and protein degradation processes. Proteolysis in skeletal muscle is closely regulated by ubiquitin ligases. In skeletal muscle, Cullin-RING ubiquitin ligases comprise the largest known category of ubiquitin ligases as shown in Table 1. Cullin-RING ubiquitin ligases regulate various cellular processes, including multiple aspects of the cell proliferation, transcription, signal transduction, and development [28]. Especially, the muscle-specific E3 ubiquitin ligases, such as the muscle RING finger 1 (MuRF1) and the muscle atrophy F-Box (Atrogin-1/MAFbx), are involved in the regulation of protein degradation in skeletal muscle [29–31]. The expression of these two ubiquitin ligases has been shown to markedly increase in skeletal muscle atrophy [32, 33]. Studies have suggested that titin, Myosin Heavy Chains, Myosin Light Chain-1/2 as the substrates of MuRF1 and elongation initiation factor 3 subunit f (eIF3-f), MyoD, and myogenin as the substrates of Atrogin-1/MAFbx [34]. Although their specific substrate in vivo is yet to be elucidated, MuRF1 or Atrogin-1/MAFbx knockout mice are resistant to muscle atrophy induced by denervation [32], suggesting that these two genes are important regulators of muscle atrophy.

**Table 1:** Ubiquitin ligases related with UPS in skeletal muscles modified from Ref. [82]

Ubiquitin ligases	Target or affected proteins	Refs.
MuRF1 (TRIM63)	Sarcomeric proteins, myosin-binding protein (MYBPC1), troponin 3, telethonin	[83–86]
MuRF3 (TRIM 54)	Sarcomeric proteins, filamin	[83, 87]
TRIM32	Actin, desmin	[88]
MUSA1	–	[37]
SMART	–	[37, 38]
Nedd4	MTMR4, FGFR1, Notch 1	[89, 90]
TRAF6	Ubc13, K63-linked ubiquitination	[128–130]
Cullin adaptors		
Atrogin-1/MAFbx	Actin, titin, calsarcin-1, MYHBPC3	[91, 92]
Cbl-b	Insulin receptor substrate 1 (IRS-1)	[35, 36]
KLHL40	Filament protein	[95]
KBTBD13	Z-disc proteins	[96]
KLHL41	Nebulin, nebulin-related anchoring protein	[97–99]
KLHL20	Autophagy-related protein 13	[100, 101]

MuRF1: muscle RING finger 1; MuRF3: muscle RING finger 3; TRAF6: tumour necrosis factor receptor-associated factor 6; Cbl-b: Casitas B-lineage lymphoma proto-oncogene-b; KLHL40: Kelch-like protein 40; KLHL41: Kelch-like protein 41; KLHL20: Kelch-like protein 20

In addition to MuRF1 and Atrogin-1/MAFbx, Casitas B-lineage lymphoma proto-oncogene-b (Cbl-b) is also a known E3-ligase related to muscle atrophy. Unloading or spaceflight promotes the expression of Cbl-b; which targets IRS-1, an intermediate of IGF-I signalling which induces protein synthesis in muscle. Thus, it seems Cbl-b, at least partly, works as mechanosensing-mediated muscle atrophy [35, 36]. There are E3-ligases associated with denervation-induced muscle atrophy: muscle ubiquitin ligase of SCF complex atrophy-1 (MUSA1) and specific for muscle atrophy and regulated by transcription (SMART) [37, 38]. Moreover, Hughes et al. recently reported that an E3-ligase, F-box and leucine-rich protein 22 (Fbxl22) mediates neural inactivity-induced muscle atrophy [39]. Knockdown of Fbxl22 in denervated muscle resulted in significant muscle sparing. The expression of ubiquitin ligase is regulated by a transcription factor, Forkhead box O (FoxO) [30]. Akt phosphorylates FoxOs, thereby resulting in their export from the nucleus to the cytoplasm. On the other hand, when the Akt pathway is attenuated by models of muscle atrophy, FoxOs are imported to the nucleus and induce the expression of ubiquitin ligases. Although most of the E3-ligases remain to be explored in the context of muscle atrophy, these findings indicate that the UPS plays a substantial role in muscle atrophies.

### UPS dysfunction causes skeletal muscle atrophy

The UPS degrades most of the long- and short-lived normal as well as abnormal intracellular proteins [40]. Especially in the muscle, most of the myofibrillar proteins are degraded through the UPS [41, 42]. We generated muscle-specific Rpt3-knockout mice (Mlc1f-Cre;Rpt3^f/f^) to better understand the role of the proteasomal system in the skeletal muscle tissue. The proteasomal subunit Rpt3 deletion significantly decreases protease activities and increases ubiquitinated proteins [14]. The muscle-specific deletion of Rpt3 resulted in reduced physical activity, and a decrease in the force production in mice, accompanied with the accumulation of abnormal proteins [14]. In addition, in muscle-specific Rpt3-deficient mice, muscle weight divided by body weight was significantly smaller in the gastrocnemius and tibialis anterior muscles, which are predominantly type II fibres, but not in the soleus muscle, which is predominantly type I fibres [14]. Previous studies have reported that type II glycolytic muscle fibres are more susceptible to muscle wasting conditions than are type I oxidative fibres [43, 44]. Since genetic induction of PGC-1α shows resistance to atrophy [45], the pro-atrophic response in type II fibres may be partly due to a low content of PGC1α. Interestingly, the proteasome-deficient mice showed premature death [14], where it further shows the importance of skeletal muscle homeostasis maintained by the UPS.

### Crosstalk with autophagy system in muscle-specific proteasome dysfunction

Another degradation process, which is known as autophagy is the natural regulatory cellular mechanism that is mainly involved in the removal of unnecessary or dysfunctional components from the cell [46]. Autophagic process initiates by forming a flat membrane cistern that envelops a portion of cytoplasm, eventually forming a closed double-membrane vesicle, which is known as the autophagosome. The autophagosome further fuses with the lysosome where its cargo components are degraded. Although autophagy is marginally activated in basal conditions, main factor responsible for the activation is the nutrient starvation [46, 47]. It has also been shown that mice deficient in Atg3, Atg5, or Atg7, which are involved in autophagy, respectively, appear almost normal at birth but die on the first day of birth [48–50].

Autophagy is also shown to be essential for the maintenance of the skeletal muscle homeostasis. With regard to the skeletal muscle, the excessive activation of autophagy promotes muscle wasting [51–53]. Conversely, the muscle-specific deletion of a crucial autophagy gene, Atg7, results in profound muscle atrophy and age-dependent decrease in the generation of muscle force [54]. Furthermore, when mTORC1 was constitutively activated in skeletal muscle by TSC1-knockout, autophagy is inhibited, and results in late onset severe myopathy [55]. Thus, autophagy plays an important role in muscle plasticity and homeostasis.

Since the UPS and autophagy pathways are differently oriented, these two proteolysis systems were viewed as independent [56, 57]. However, there are emerging evidences suggesting the occurrence of a crosstalk between the autophagy and proteasomal pathways in the skeletal muscle. Although autophagy had been thought as a non-specific degradation system, it was found to degrade ubiquitinated proteins [58]. Moreover, muscle-specific autophagy dysfunction induces an increase in ubiquitinated proteins during denervation [54], which would be compensatory upregulation of UPS. Our previous study [14] also suggests the autophagy–UPS complementary relationship. Proteasome-deficient mice exhibited the activation of autophagy. Briefly, the protein levels of LC3II, a standard marker for autophagosomes, and ubiquitin-binding p62 were found to be increased in the Rpt3−/− gastrocnemius muscle. Moreover, the levels of Beclin-1 and Atg5 that are involved in the formation of the isolation membrane, and LC3I, which is involved in the initiation of autophagosome formation, were increased in the Rpt3−/− muscle. Therefore, the autophagy pathway seems to be enhanced in the UPS-deficient muscle. Previous studies also have demonstrated that inhibition of the UPS induces autophagy in vivo and in vitro [59, 60]. Taken together, the UPS and autophagy systems, at least in part, compensatory maintain myocellular homeostasis and integrity.

### UPS in muscle stem cells and regeneration

**Fig. 2:**
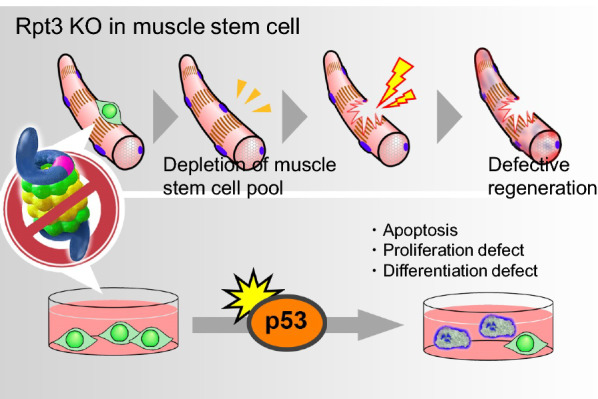
Proteasome dysfunction in Rpt3-deficient muscle stem cells impaired their ability to proliferate, survive and differentiate, resulting in defective muscle regeneration. We also found that proteasome inactivation by Rpt3 deficiency in primary myoblasts inhibits cell proliferation and induces apoptosis. Further, proteasome dysfunction conferred by satellite cell-specific Rpt3-knockout induces p53 activation

The adult muscle stem cells (also known as satellite cells) are required for the regeneration of the adult skeletal muscle [61]. Responding to muscle damage, satellite cells are rapidly activated and start to proliferate. Three days after the cardiotoxin injection-induced muscle damage, when the satellite cells are under the process of muscle regeneration, we found that both chymotrypsin-like and trypsin-like proteasome activities were increased. Using satellite cell-specific proteasome-deficient mice (Pax7-CreERT2; Rpt3^f/f^), we demonstrated that proteasome dysfunction impaired satellite cell ability to proliferate, survive, and differentiate, resulting in defective muscle regeneration [62] (Fig. 2). These findings indicate that the UPS activity in satellite cell is associated with muscle regeneration, especially in the early phase [62]. Consistently, in a previous study, proteasome activity was found to be strongly correlated with proliferating cell nuclear antigen protein levels, suggesting that proteasomes play a key role in satellite cell proliferation [63]. Therefore, the findings suggest that the enhancement of the proteasome system is important for satellite cell proliferation and normal muscle regeneration.

Previous studies have also reported that the UPS is associated with the process of myogenic differentiation of satellite cells, which begins with cell cycle arrest and ends with the fusion of individual cells to form multinucleated myotubes [64–66]. In addition, it has also been shown that the inhibition or knockdown of the proteasome can block the fusion of myoblasts and thereby inhibit the process of differentiation [65, 66].

A lot of uncertainty still remains, regarding the relationship between the UPS and muscle regeneration. One possible candidate to explain the relationship would be p53, which regulates apoptosis and cell survival, and promotes muscle differentiation in myoblasts [67–71]. A recent study reported that the tight control of p53 levels in myoblasts regulates the balance between the differentiation process and the return to the quiescence stage [72], which indicates the importance of the p53 during the process of myogenic differentiation. Interestingly, p53 was upregulated in Rpt3-deficient primary satellite cells and the inhibition of p53 expression by siRNA in those cells reduced cell death [62]. These data indicate that Rpt3 in satellite cells is important for cell survival via p53. There is still ambiguity with regard to muscle regeneration and the UPS. Although further work needs to be carried out to understand the UPS, recent studies have revealed a link between the UPS and various diseases discussed the following section.

### Proteasome system and related diseases

As the UPS is important for the maintenance of the homeostasis in skeletal muscle, dysfunctions in the system may lead to pathological conditions such as muscular dystrophy, myositis, cachexia and amyotrophic lateral sclerosis.

### Diseases with disorganisation of skeletal muscle itself

#### Muscular dystrophy: aberrant developmental process

Dystrophin, a 427-kDa skeletal muscle protein, links the interior of the myofibres to the extracellular matrix. Mutations in the dystrophin gene are linked to a severe form of muscular dystrophy known as Duchenne muscular dystrophy (DMD) or a mild form known as Becker muscular dystrophy (BMD) [73]. Dystrophin missense mutations cause a wide range of severe phenotypical features in DMD patients. When these missense mutations are stably expressed in mammalian myoblasts, dystrophin proteins are unstable and the protein levels are decreased by proteasomal degradation, but still mutated dystrophin functions to some extent [74]. Continuous administration of the proteasomal inhibitor MG-132 effectively rescues the plasma membrane localisation and the amount of dystrophin in skeletal muscle fibres in dystrophin-deficient model mice [75]. Proteasomal inhibitors have been shown to reduce muscle membrane damage, as revealed by vital staining of the diaphragm and gastrocnemius muscle isolated from treated mice with dystrophin deficiency [75]. Bortezomib, which is a proteasome inhibitor, restores dystrophin and dystrophin–glycoprotein complex at the sarcolemma, which improves the dystrophic phenotype [76]. Compared with control animals, muscular dystrophy dogs treated with bortezomib had lower amount of connective tissue deposition and inflammation as evidenced by muscle histology, collagen morphometry, and ultrastructural microscopy [77].

Congenital muscular dystrophy, which is caused by mutations in the gene encoding the laminin α2 chain, is a severe and incapacitating disease [78]. Proteasome activity is increased in laminin α2 chain-deficient muscle, and treatment with MG-132 reduces muscle pathology in laminin α2 chain-deficient mice [78]. In addition, bortezomib reduces proteasome activity in congenital muscular dystrophy type 1A myoblasts and myotubes [78]. Hence, proteasome inhibition might be useful in patients lacking the laminin α2 chain as a supportive treatment [79]. In case of dysferlinopathy, it was reported that proteasomal inhibition restores biological function of missense mutated dysferlin in patient-derived cells, but the paper was retracted [80]. The effect of proteasome inhibition in muscular dystrophy is yet to be elucidated.

Overall, proteasome inhibition might block the degradation of the mutant dystrophin and recover the function to some extent.

#### Disease related to mutants in the proteins of UPS system: abnormal maintenance of muscle structures

Protein aggregate myopathies are characterised by protein accumulation in myofibres. These myofibres contain inclusions of sarcomere proteins including myosin and myosin-associated proteins with aberrantly distributed microtubules [81]. Since sarcomere proteins are degraded by UPS, UPS dysfunction might be central to the development of these diseases. It is important to understand how genetic mutations of UPS impact on sarcomere integrity.

Several disease-causing mutations have been found in ubiquitin E3-ligase and its adaptors (Table1) [82]. The genes tripartite motif-containing 63 and 54 (TRIM63 and TRIM54) encoding MuRF1 and MuRF3, respectively, are RING ubiquitin ligases involved in UPS. Both MuRFs are microtubule-associated proteins located in the M bands and Z discs of the sarcomere [83]. These E3 ubiquitin ligases play a role in the degradation of sarcomeric proteins, myogenesis, and stabilisation of microtubules. The targets of MuRF1 include myosin-binding protein (MYBPC1), troponin 3, and telethonin, which cause distal arthrogryposis-1B myopathy [84], dilated cardiomyopathy-2A [85], and limb–girdle muscular dystrophy (LGMD)-7 [86], respectively. MuRF3 targets filamin, and MuRF3 mutations are associated with distal myopathy, myofibrillar myopathy, and restrictive cardiomyopathy-5 [87]. Another TRIM protein, TRIM32, targets actin, and desmin. A mutation in TRIM32 results in LGMD, nemaline myopathy, or myofibrillar myopathy [88]. The ubiquitin ligase Nedd4 participates in denervation-induced muscle atrophy in mice [89], and a relationship between myotonic dystrophy type 2 and NEDD4 has also been reported in human [90]. Atrogin-1/MAFbx is the Cullin adaptor of UPS. Atrogin-1 targets actin and disconnects actin, titin, and calsarcin-1 [91]. Atrogin-1 also targets MYHBPC3, and mutation in the gene results in dilated cardiomyopathy [92].

The Kelch-like family members, the adaptor proteins of E3-ligase, are mutated in nemaline myopathy [93]. Mutations in the Kelch family affect Cullin 3 (CUL3) interaction, which in turn affects the ubiquitination and degradation of protein substrates targeted by CUL3 protein complex [94]. Several genes among the Kelch-like family members are involved in nemaline myopathy; Kelch-like protein 40 (KLHL40) decreases thin filament protein stability [95], KBTBD13 disorganises the Z-disc [96], and Kelch-like protein 41 (KLHL41) impacts on nebulin degradation [97, 98]. KLHL41 degrades nebulin-related anchoring protein, and this degradation is dysregulated in nemaline myopathy [99]. Especially, the CUL3 and KLHL20 complex coordinates the amount of autophagy-related protein 13 (ATG13) in prolonged starvation and controls autophagy [100, 101].

Overall, mutations in several E3-ligases can be the cause of muscle diseases. Ubiquitin ligases could mediate the interplay between autophagy and ubiquitin proteasomal degradation.

#### Cachexia: reduced stress tolerance of the skeletal muscle

Cachexia reflects muscle wasting syndromes associated with several chronic diseases, such as cancer, diabetes, chronic obstructive pulmonary disease, congestive heart failure and chronic kidney disease (CKD) [102]. Body weight loss, muscle wasting, adipose tissue depletion and abnormal metabolism are among the characteristics of cachexia [103]. While the underlying mechanisms are complex, elevated angiotensin II (Ang II) levels are frequently found in patients with cachexia, and treatment with angiotensin-converting enzyme inhibitor prevents weight loss [104]. Diaphragm muscle biopsies from 22 critically ill patients under mechanical ventilation appeared approximately 25% smaller in myofibre diameter, and this reduced their contractile force by one-half or more [105]. This protein degradation is mediated by proteolytic pathways, including proteasome and lysosomes [103]. The expression levels of muscle-specific ubiquitin ligases, such as Atrogin-1/MAFbx, MuRF1/TRIM63, SMART [38], have been accepted as molecular markers of enhanced proteasome-dependent proteolysis in cancer-related cachexia demonstrated in several types of experimental models [106]. Small-molecule inhibiting MuRF1 attenuates skeletal muscle atrophy in cardiac cachexia mouse model [107]. Sestrin1, which is a stress-inducible metabolic regulator, preserves muscle mass and force in atrophy condition mouse models, including sarcopenia by blunting FoxO-dependent atrogenes [108]. On the other hand, specific proteasome inhibitors such as bortezomib do not improve the muscle phenotype in cancer-associated cachexia [109]. Others have reported unchanged levels of the UPS pathway in cancer patients [110, 111].

Depending on the type of cachexia pathology, there could be different protein degradation pathways involved. Interventional studies using cachexic models are needed to elucidate the role of UPS in the cachexic state.

### Diseases characterised with inflammations

#### Nakajo–Nishimura syndrome

Proteasome-associated auto-inflammatory syndromes was firstly described in 1939 in patients presenting with recurrent fever beginning in early childhood, accompanied by nodular erythema, rash, and joint contractures [112]. Since then, several syndromes, such as the chronic atypical neutrophilic dermatosis with lipodystrophy and elevated temperatures (CANDLE) syndrome, the Nakajo–Nishimura syndrome (NNS), Joint contractures/Muscle atrophy/microcytic anaemia/the Panniculitis-induced lipodystrophy (JMP) syndrome, and the Japanese auto-inflammatory syndrome with lipodystrophy, have been used to categorise patients with diseases within the same spectrum, including myositis [113, 114]. As mentioned in Sect. 2, an association between the immunoproteasome and disease has also been reported. Independent studies identified mutations in the immunoproteasome subunit β5i gene that result in a sustained inflammatory response [113, 115]. In response to cytokines, DNA damage, or oxidative stress, cells selectively upregulate the expression of the immunoproteasome. Immunoproteasome accelerates the proteolysis of specific peptide substrates and allows for the facilitated degradation of oxidant damaged proteins, which may accumulate during inflammation. Immunoproteasome-mediated proteolysis generates immunogenic epitopes presented by MHC class I molecules [116].

Immunoproteasomes are also critical for skeletal muscle differentiation of myoblasts [117]. The oxidative pathway is dysregulated in β5i-mutated iPS cell-induced myeloid cells [118]. In a DMD animal model, inhibition of the immunoproteasome was reported to ameliorate cardiomyopathy in mdx mice, reducing the fibrosis [119, 120]. The mechanism of β5i mutations in skeletal muscles remains to be elucidated. These studies are enhancing the importance of analysing about immunoproteasome in the disease mechanism of NNS and muscular dystrophy.

#### Sporadic inclusion body myositis

Sporadic inclusion body myositis (sIBM) is the most common form of inflammatory myopathy in individuals aged 50 years or more [121, 122]. Muscle weakness apparent in the quadriceps, wrist flexors, and finger flexors are the typical clinical findings of sIBM [123]. A muscle biopsy would typically reveal endomysial inflammation, invasion of mononuclear cells into non-necrotic fibres, and rimmed vacuoles. These pathological hallmarks indicate that both inflammation and degeneration contribute to the pathology. As such, sIBM is considered to be caused by protein unfolding/misfolding combined with the formation of inclusion bodies [124]. Inhibition of the elimination of ubiquitinated misfolded/unfolded proteins by proteasome results in cellular accumulation of protein aggregates found in sIBM-affected muscle fibres [14]. Amyloid beta (Aβ) and phosphorylated tau (p-tau) can be found in these aggregates. The effect of Aβ precursor protein (APP) overexpression on proteasome function and the influence of proteasome inhibition on aggresome formation was examined using cultured human muscle fibres [125]. In sIBM-affected muscle biopsies, the 26S proteasome subunits have been immune-detected in the β-tubulin-associated aggresomes, which also contained Aβ, p-tau, ubiquitin, and heat shock protein 70 (HSP70). Cultured muscle fibres have been observed to overexpress APP and display diminished proteasomal proteolytic activity, and the addition of a proteasome inhibitor strikingly increases aggresome formation apparently. The formation of inclusion bodies might be followed by abnormal intracellular accumulation of unfolded proteins [126]. As mentioned in Sect. 3.3, the crosstalk between proteasome and autophagy is important. Recently, the role of chaperone-mediated autophagy in the aetiology of sIBM was investigated [127]. Several ubiquitin proteasome genes modulate autophagy. For example, Beclin-1, an essential gene for activation of autophagy, is ubiquitinated by TNF receptor-associated factor 6 (TRAF6) ligase [128]. In addition, TRAF6 has been reported to be involved in muscle atrophy in several cases [128–130].

Dysfunction of UPS, which results in an enhanced autophagic machinery, may be the cause of muscle atrophy in sIBM. Accordingly, in contrast to the protective effect of proteasome inhibitor in muscular dystrophy, proteasome dysfunction may play a role in the accumulation of misfolded, potentially cytotoxic proteins in sIBM myofibres.

### Motor neuron disease: indirect cause of muscle atrophy

Amyotrophic lateral sclerosis (ALS) is a neurodegenerative disorder characterised by the fatal progressive loss of upper and lower motor neurons. In particular, the accumulation of ubiquitinated inclusions containing ALS-causing gene products is a common feature in most familial ALS models; it is also a pathological hallmark of sporadic ALS, indicating that the failure to eliminate detrimental proteins is linked to the pathogenesis of both sporadic and familial ALS. The involvement of UPS dysfunction is strongly suggested by the presence of ubiquitinated inclusions such as skein-like and round hyaline inclusions [131]. The proteasome system is dysregulated in ALS, and the accumulation of superoxide dismutase 1 (SOD1) deposits can be found in the spinal cord of experimental animals and human autopsy cases [132–134]. The continuous expression of mutant SOD1 decreases proteasome activity, and primary cultured embryonic motor neurons are vulnerable to proteasome inhibitor [134]. Proteasome subunit Rpt3 conditional knockout mouse in a motor neuron-specific system showed locomotor dysfunction accompanied by progressive motor neuron loss and gliosis [135]. Promoting proteasomal degradation could be a therapeutic strategy for ALS [136].

Mutation causing ALS can also affect the function of UPS. Ubiquilin-2 and p62, two disease-causing genes in ALS, are mainly related to the protein aggregation and degradation pathways; therefore, mutations in the ubiquilin-2 and p62 genes can cause ALS related disorders [137–139]. Recently, exome sequencing of the ALS-FTD family identified the CCNF (encoding cyclin-F) gene as a novel gene associated with ALS [140]. Cyclin-F is the part of Skp1-Cul1-F-box (SCF) E3 ubiquitin-ligase complex that enables proteasome degradation [141]. Cyclin-F binds to valosin-containing protein (VCP), which is also reported to be mutated in ALS [142]. The ATPase activity of VCP promotes cytoplasmic aggregation of TAR DNA-binding protein 43 (TDP-43), which is commonly observed in degenerating neurons in ALS patients [142]. The inhibition of proteasome in motor neuron can be the cause of aggregation of SOD1 or TDP-43, which would be involved in ALS pathomechanism.

Overall, in case of motor neuron disease, UPS has a role to eliminate pathologically aggregated proteins rather than preserving the amounts of functional molecules as is in the case of structural proteins in muscular dystrophy. The inhibition of proteasome in motor neuron can be the cause of aggregation of SOD1 or TDP-43, which would be involved in ALS pathomechanism.

## Conclusions

In skeletal muscle, a functional decline due to atrophy is regulated by proteolysis. This nature of the tissue makes the relationship between proteolysis and skeletal muscle complicated. Indeed, the UPS inhibition often leads muscle atrophy and deficit in regeneration, while preserving skeletal muscle in some conditions or diseases. The proteasome inhibitors and ubiquitin ligases are important regulators of the proteolytic system and may be potential therapeutic targets. Further studies are needed to understand how the UPS regulates the dynamics of proteostasis in skeletal muscle, and how its aberrance/dysfunction induces the pathological state. The study considerably enhances our understanding of the UPS regulation/maintenance of skeletal muscle. It will thus be of immense importance to further elucidate the mechanisms behind proteasome-mediated proteolysis, which will ultimately allow us to develop therapeutic intervention for the related diseases.

## Data Availability

Not applicable.
